# Supplementary material for: Netcombin: An algorithm for constructing optimal phylogenetic network from rooted triplets
Source: PLoS One. 2020 Sep 18;15(9):e0227842. doi: 10.1371/journal.pone.0227842 (PMC7500971; doi:10.1371/journal.pone.0227842)
Supplement: S4 File — (ZIP) [file pone.0227842.s004.zip › SequenceData-Triplet/sequenceData-10-20-30-0-Triplet/length 1000, numseq 40, rate 10^-7/2 seq.pdf]

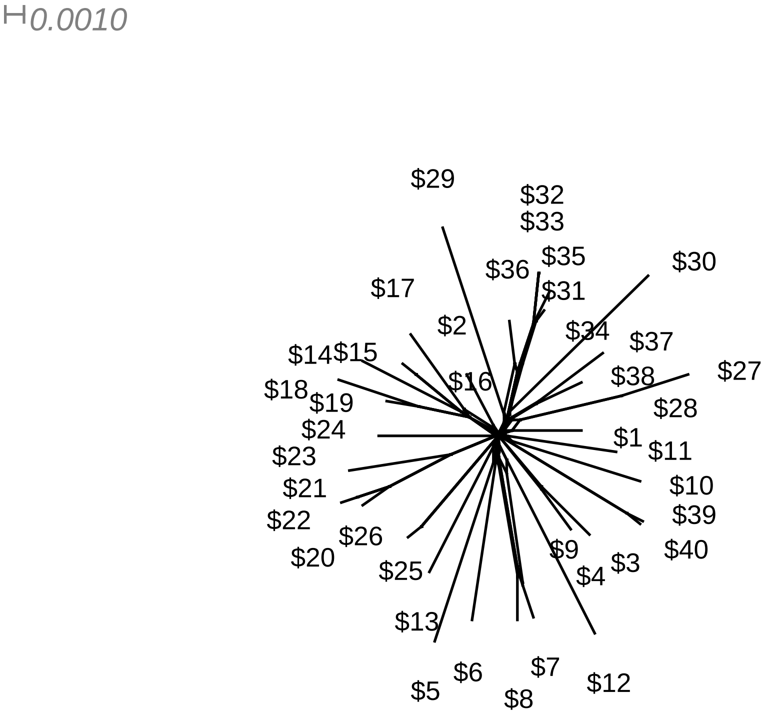
0.0010
$29
$32
$33
$35
$36
$30
$31
$17
$2
$34
$37
$14
$15
$27
$38
$16
$18
$19
$28
$24
$1
$11
$23
$10
$21
$39
$22
$26
$40
$9
$3
$20
$4
$25
$13
$7
$6
$12
$5
$8
